# Bilateral synchronous occurrence of three different histological types of renal tumor: a case report

**DOI:** 10.1186/1752-1947-3-6798

**Published:** 2009-04-01

**Authors:** Demetrios Radopoulos, Anastasios Tahmatzopoulos, Nikolaos Kalinderis, Georgios Dimitriadis

**Affiliations:** 1 1st Department of Urology, Aristotle University of Thessaloniki, Ethnikis Amynis 41, 54635 Thessaloniki, Greece

## Abstract

**Introduction:**

Renal cell carcinomas account for 85% of all renal neoplasms. With the introduction of modern imaging modalities, there has been an increased diagnosis of renal tumors. Recent studies have shown that partial nephrectomy can be as safe as radical nephrectomy for smaller renal tumors. Renal cell carcinomas are usually unilateral, however, they can be bilateral in 2% to 4% of sporadic cases and considerably more common in familial cases.

**Case presentation:**

In this case report, we describe an unusual case of two bilateral synchronous chromophobe renal cell carcinomas accompanied by an oncocytoma and an angiomyolipoma, that were all treated by open partial nephrectomy.

**Conclusions:**

To the best of our knowledge, this is the first case report on the synchronous occurrence of bilateral chromophobe renal cell carcinomas associated with an oncocytoma and an angiomyolipoma.

## Introduction

Renal cell carcinoma (RCC) accounts for 85% of all renal neoplasms. Its incidence has been rising due to the increased use of ultrasonography and computed tomography (CT) scans for the evaluation of patients with a diversity of presenting symptoms. Most renal cell carcinomas are unilateral, but bilateral tumors, synchronous or asynchronous, have been found in 2% to 4% of reported sporadic cases. However, the incidence is higher among patients suffering from Von Hippel-Lindau (VHL) disease and other familial cases.

Radical nephrectomy is considered the standard treatment modality for renal cell carcinomas. However, recent data have shown that partial nephrectomy is as safe and effective as radical nephrectomy for tumors smaller than 4 cm, and more recent studies support the fact that the indications for partial nephrectomy can be safely extended to tumors up to 7cm.

In this case report, we describe an unusual sporadic case of bilateral synchronous RCC accompanied by an oncocytoma and an angiomyolipoma that were all treated by open partial nephrectomy.

## Case presentation

In February 2006, a 57-year-old man presented with vague right upper quadrant discomfort. He had no surgical history and was on antihypertensive (perindopril) and antidiabetic (metformin) medication. He had a history of acute myocardial infarction 5 years earlier and was suffering from hypertensive cardiomyopathy. He was subjected to abdominal ultrasound which revealed bilateral renal tumors, and cholelithiasis which was probably the cause of his right upper quadrant discomfort.

In order to investigate the finding of bilateral renal tumors, a contrast CT scan was performed which showed two round hyperdense masses, one in each kidney, with homogeneous contrast uptake and well defined margins, arising from the renal cortex. The differential diagnosis included lymphoma, atypical cysts, metastases and RCC.

**Figure 1:**
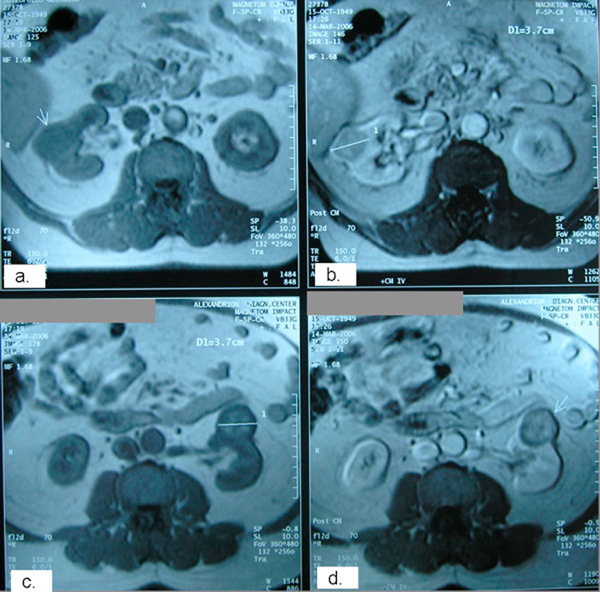
**Pre-operative axial magnetic resonance imaging sections showing a 3**.7 cm tumor arising from the middle of the right kidney (**a, b**: post contrast) as well as a 3.7 cm tumor arising from the anterior surface of the left kidney (**c, d**: post contrast).

**Figure 2:**
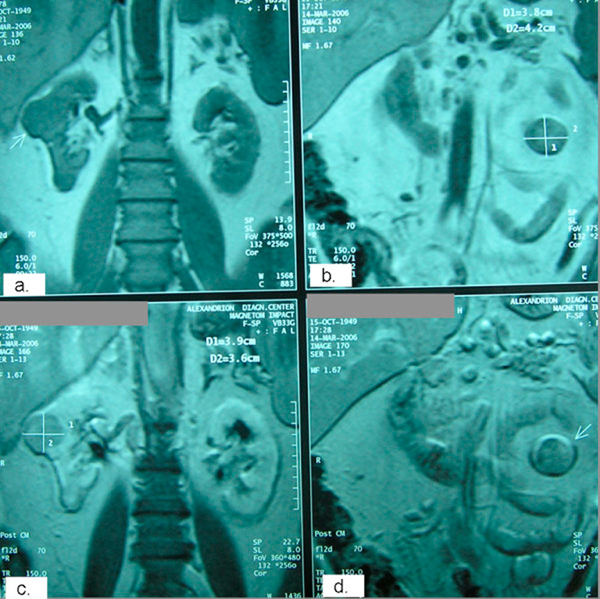
**Pre-operative coronal magnetic resonance imaging sections demonstrating the aforementioned right (**a, c**: post contrast) and left (**b, d**: post contrast) renal tumors**.

Subsequent magnetic resonance imaging (MRI) confirmed an enhancing, exophytic, well circumscribed solid tumor, arising from the middle of the right kidney (3.7cm). On the anterior surface of the left kidney, an enhancing, exophytic, well circumscribed solid tumor was described (Figures 1 and 2) and no abnormally enlarged retroperitoneal lymph nodes were detected. Bone scan with ^99m^Tc-MDP did not show evidence of bone metastases.

In April 2006, the patient was subjected to open partial nephrectomy on the left kidney, which revealed a 4 cm chromophobe renal cell carcinoma (Fuhrman II), with clear surgical margins.

**Figure 3:**
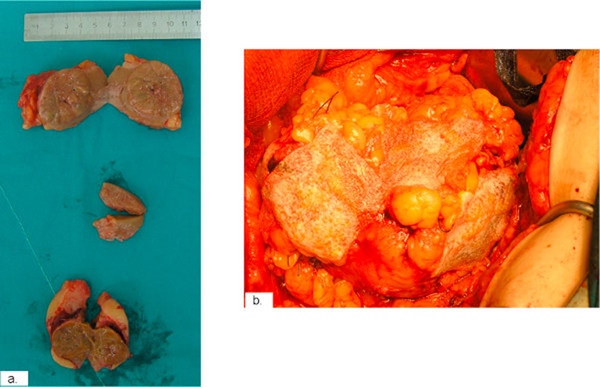
**Intra-operative images showing **(a)** removal of three renal tumors in the right kidney as well as **(b)** intra-operative use of a surgical collagen sponge containing the coagulation factors fibrinogen and thrombin**.

In May 2006, a second open partial nephrectomy was performed on the right kidney, revealing a 3.5 cm chromophobe renal cell carcinoma (Fuhrman II), with clear surgical margins. A 3 cm oncocytoma and a 1.5 cm angiomyolipoma were also detected intra-operatively, necessitating two further partial nephrectomies on the right kidney (Figure 3). A surgical collagen sponge with fibrinogen and thrombin was used to aid in hemostasis in both kidneys.

**Figure 4:**
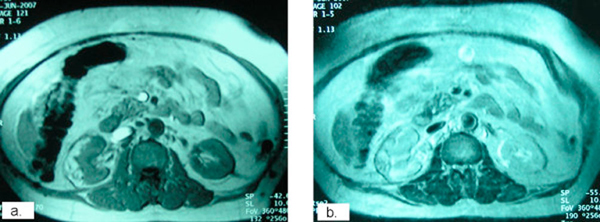
**Follow-up axial magnetic resonance imaging sections at 14 months postoperatively showing scar formation without local recurrence (**a, b**: post contrast)**.

The patient did well postoperatively. Follow-up MRI at 14 months showed no evidence of recurrence (Figure 4).

## Discussion

According to the literature, the synchronous presentation of renal tumors of diverse histology and dignity is a rare phenomenon [1,2]. The majority of published cases describe the coincidence of angiomyolipomas and renal cell carcinoma in otherwise healthy individuals as well as in tuberous sclerosis (TS) patients. The latter is an autosomal-dominant disorder characterized by mental retardation, epilepsy, and adenoma sebaceum, a distinctive skin lesion [1]–[5]. A few interesting reports describe the coincidence of RCCs and oncocytomas in patients with Birt-Hogg-Dube syndrome (BHDS), which is characterized by the development of fibrofolliculomas, renal tumors and spontaneous pneumothorax [6,7]. Our patient had no known family history of renal tumors, nor did he have clinical signs suggestive of either TS or BHDS. The patient had no clinical evidence of VHL disease (no retinal angioma on ophthalmologic examination, no family history, no epididymal or pancreatic cysts, no pheochromocytoma). Also, RCC in VHL patients is usually clear cell, whereas our patient had chromophobe RCC. Taking these facts into account, we decided not to proceed with genetic analysis of the specimen.

Oncocytomas are the most common benign renal tumors (3% to 7% of all solid renal tumors) [8]. They are usually diagnosed incidentally during routine ultrasound examination and the discrimination between RCC and oncocytoma based solely on radiologic criteria, including CT and/or MRI, is not always possible. Therefore, aggressive surgical intervention is almost always warranted [9].

Angiomyolipoma (AML) is a benign clonal neoplasm that consists of varying amounts of mature adipose tissue, smooth muscle, and thick-walled vessels [10]. Approximately 20% of AMLs are found in patients with tuberous sclerosis [11]. Patients typically present with abdominal or lumbar pain as a result of massive retroperitoneal hemorrhage and/or a palpable abdominal mass. The presence of fat within a renal lesion on CT scan, confirmed by Hounsfield units ≤10, is considered diagnostic for AML [10].

Renal cell carcinoma is the most common malignant renal tumor and accounts for 3% of all adult malignancies. Although potential etiologic factors have been identified in animal models for example, viruses, lead compounds, and more than 100 chemicals, no specific agent has been definitively established as causative in human RCC [10]. A familial form has been identified in patients with VHL disease, a rare autosomal-dominant disorder. Manifestations include the development of RCC, pheochromocytoma, retinal angiomas, and hemangioblastomas of the brain stem, cerebellum, or spinal cord. Most RCCs are asymptomatic at the time of diagnosis. The classic triad of flank pain, gross hematuria, and palpable abdominal mass is now rarely found. Therapy is almost always surgical, either in the form of radical or simple nephrectomy or in the form of nephron sparing surgery.

## Conclusion

In this paper, we present an unusual sporadic coexistence of three different types of renal tumor. A similar case with the concurrent occurrence of three primary neoplasms in the same kidney - oncocytoma, chromophobe renal cell carcinoma and angiomyolipoma - has recently been reported [12]. It is interesting that two of the three tumors in the right kidney were missed in pre-operative imaging and were only detected intra-operatively. This suggests that caution should be exercised in the interpretation of radiological results and that the surgeon should be alert for unexpected intra-operative findings.

We need to acknowledge that the follow-up period of 14 months is limited and that the patient is still at risk of local tumor recurrence and disease progression. Chromophobe RCCs, which comprise 4% to 5% of all RCCs, have an inadequately defined clinical behavior as well as poorly defined genetic alterations. Nevertheless, the present paper underscores the feasibility of partial nephrectomy in the case of multiple bilateral renal tumors.

## Consent

Written informed consent was obtained from the patient for publication of this case report and any accompanying images. A copy of the written consent is available for review by the Editor-in-Chief of this journal.

## Competing interests

The authors declare that they have no competing interests.

## Authors' contributions

DR conceived the case report, performed the operation and drafted the manuscript. GD was involved in postoperative follow-up. AT and NK were involved in postoperative follow-up and drafted the manuscript. All authors read and approved the final manuscript.
